# Supplementary material for: Changes in Body Mass Index and Their Associations with Psychological Distress, Worries, and Emotional Eating during the COVID-19 Pandemic: A Norwegian Cohort Study
Source: Nutrients. 2023 Sep 2;15(17):3834. doi: 10.3390/nu15173834 (PMC10490145; doi:10.3390/nu15173834)
Supplement: Supplementary file 1 [file nutrients-15-03834-s001.zip › BiE study- BMI Supplementary Material.pdf]

Supplementary Material

Questionnaire items

Item focusing on emotional eating

Q: Have you, during the past week, engaged in comfort eating or eating extra in response to feeling unhappy or unsatisfied?

A: 1(never)–2–3–4–5–6–7(everyday)

Items focusing on health-related worries

Q: I have become scared and anxious (worried) that the infection will affect myself A: Not true – somewhat true – completely true

Q: I have become scared and anxious (worried) that the infection will affect one of my closest ones

A: Not true – somewhat true – completely true

Q: I have become scared and anxious (worried) that the infection will affect elderly members of my family

A: Not true – somewhat true – completely true

Items focusing on worries regarding personal economy:

Q: I fear (am worried) that the outbreak will cause me to be laid-off temporarily or lose my job

A: Not true – somewhat true – completely true

Q: I fear (am worried) that the outbreak will lead to a poorer personal economy A: Not true – somewhat true – completely true

Items focusing on consumption of high-sugary foods and drinks

Q: How often, during the past month, did you eat or drink the following

Q1: Sugar-containing soda or soft drink

A1: Rare/never – 1-3 times/month – 1-3 times/week – 4-6 times/week – daily

Q2: Candy, cakes, biscuits or sweet desserts

A2: Rare/never – 1-3 times/month – 1-3 times/week – 4-6 times/week – daily

Items focusing on consumption of fruit and vegetables

Q: During the past month, how many portions of fruit and vegetable per day did you eat often?

A: 0-1-2-3-4-5-6-7-8-9-10 or more

Items focusing on psychological distress (Hopkins symptom checklist SCL-10)

Q: Think back to the last seven days, choose the answer that applies to you. Answer as honestly as possible.

A: ‘Not at all’, ‘A little’, ‘Quite a bit’ and ‘Extremely’

1. Suddenly scared for no reason
2. Feeling fearful
3. Faintness, dizziness or weakness
4. Feeling tense or keyed up
5. Blaming yourself for things
6. Difficulties in falling asleep or staying asleep

7. Feeling blue
8. Feelings of worthlessness
9. Feeling everything is an effort
10. Feeling hopeless about the future.

Changes in psychological distress and worries over time
In the longitudinal analysis, we treated the SCL-10 score at the first time point for every participant as constant for the linear mixed model to function effectively. We then assessed the changes in SCL-10 scores over time (Table S1). The cut-off for the mean SCL-10 score was ≥ 1.85, big changes were considered ≥ 0.5 and huge changes ≥ 1. Of the participants who responded to the psychological distress items at the first, second, and/or third time point (n= 17264), around 89% did not experienced any big changes in their SCL-10 scores over time. Moreover, only around 2% had a huge change in their SCL-10 score.

Table S1. Changes in psychological distress over time

SCL-10 score over time		Frequency	Percent
**Big change**	Yes	1968	11
	No	15296	89
**Huge change**	Yes	338	2
	No	16899	98
**Total**		17264	100

We also considered the participants' answers focusing on worries at the first time point as constant over time in the longitudinal analysis. We then analyzed the changes in level of worries related to health and personal economy over time. (Table S2)

Table S2. Changes in worries over time

Changes in worries over time	Frequency	Percent
**Yes**	6069	34.5
**No**	11521	65.5
**Total**	17590	100

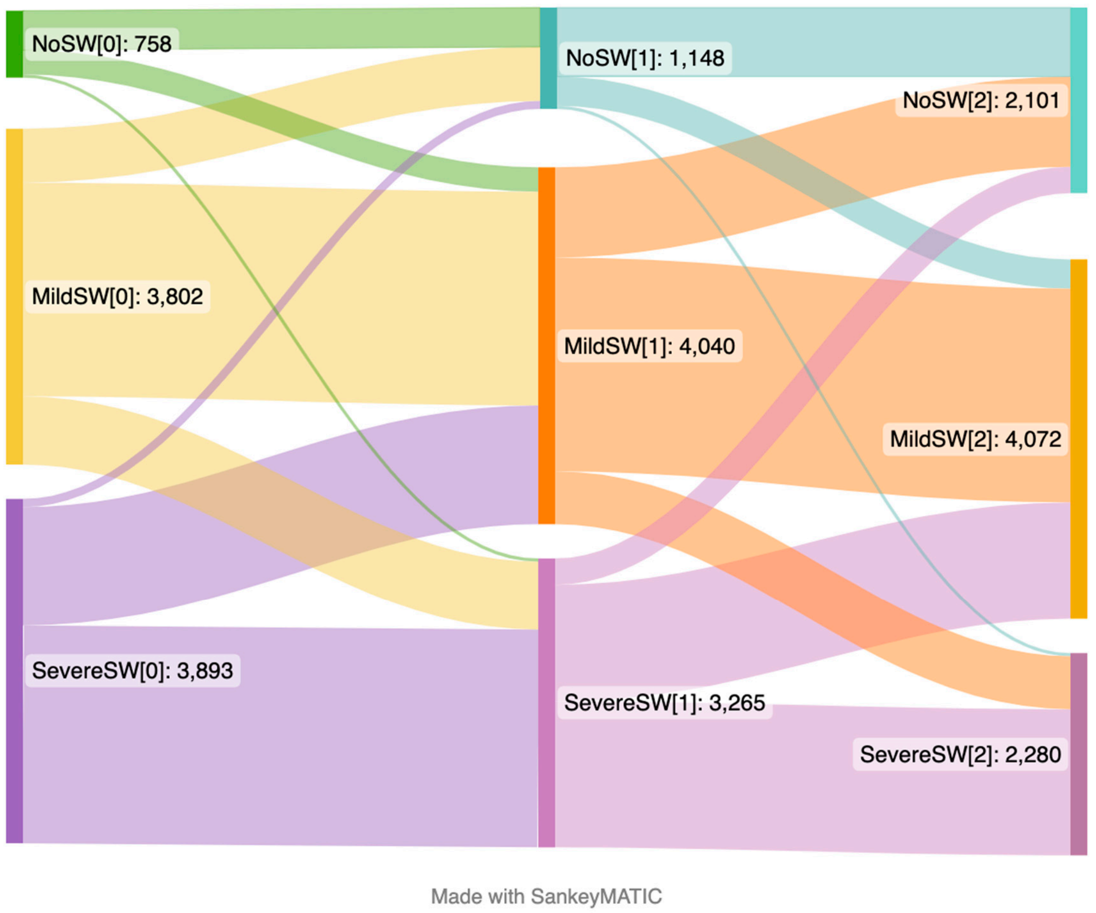

Figure S1. Sankey plot: Substantial worries over time (from the April 2020 (left side) to January 2021 (mid) and May 2022 (right side)).

This plot includes the participants that answered the items focusing on worries related to personal economy and health on the questionnaire at the first time point (t_0), second time point (t_1) and third time point (t_2) (n=8453). Those who had no worries related to health or personal economy were considered in the no substantial worry group (NoSW), those who had some worries regarding health or economy were considered in the mild substantial worry group (MildSW), and those who had either high levels of worries related to health or economy were placed in the severe substantial worry group (SevereSW). As shown in the plot, there were only minimal changes in the levels of worry from SevereSW to NoSW and vice versa. Some fluctuations are seen between time points, especially from SevereSW to MildSW; however, the overall numbers of the groups did not change considerably.

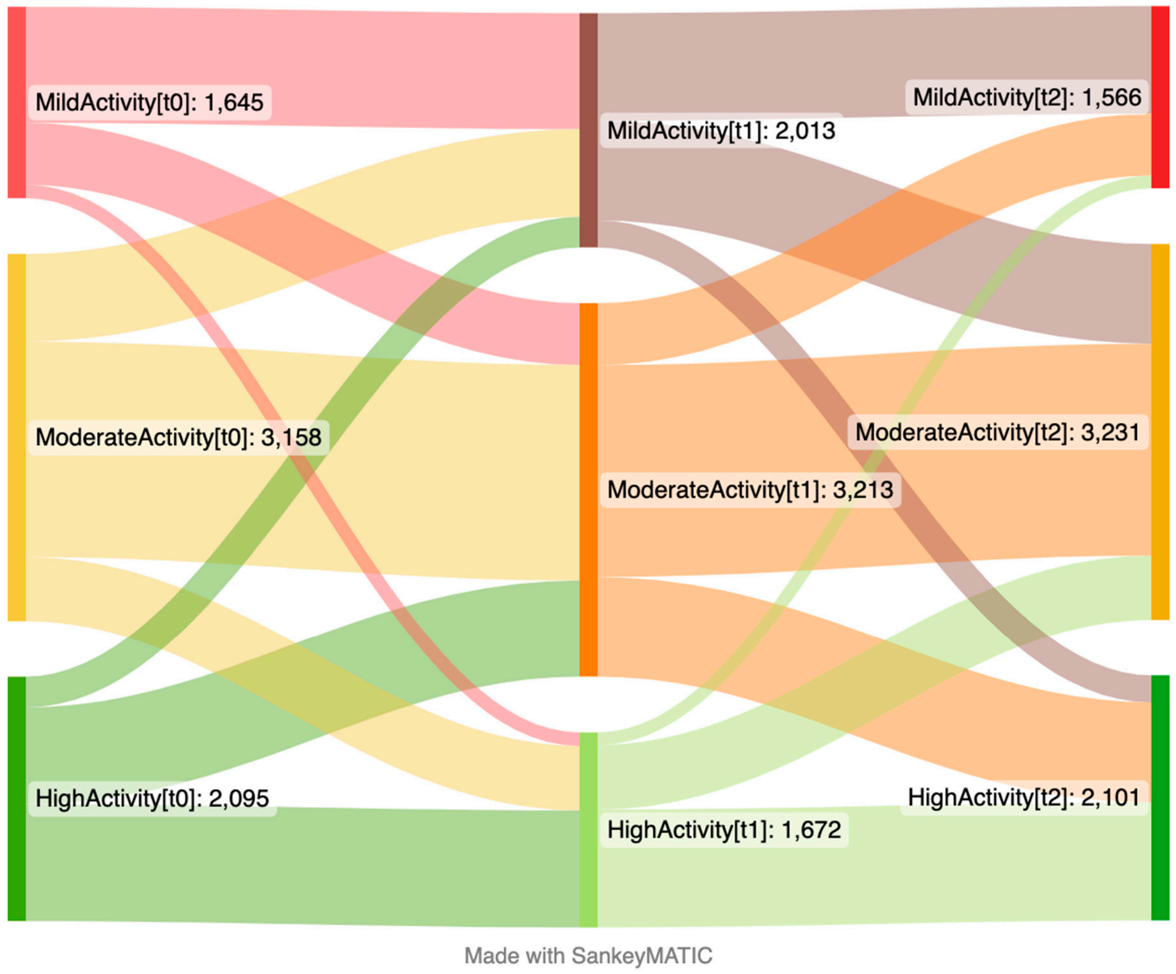

Figure S2. Sankey plot presenting changes of activity level over time (from the April 2020 (left side) to January 2021 (mid) and May 2022 (right side)). This plot includes participants answering the questions regarding activity level in all three time points (n= 6898).

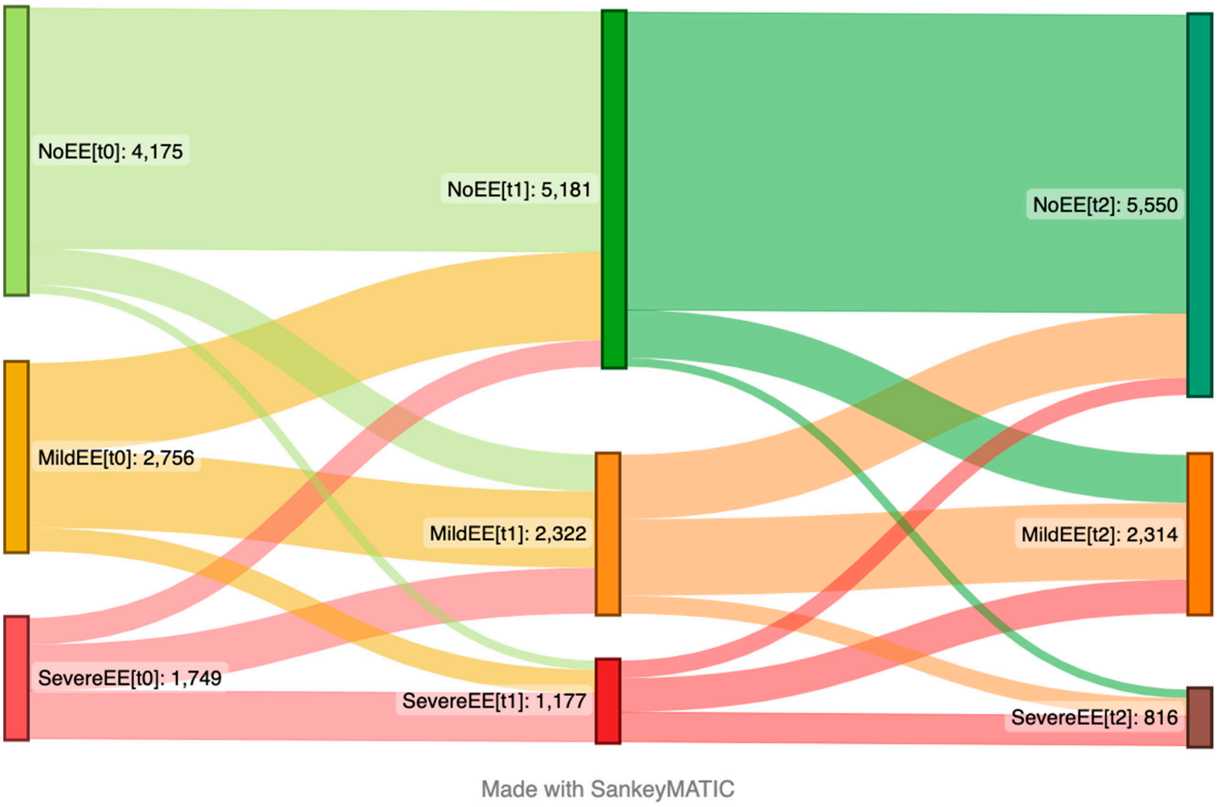

Figure S3. Sankey plot presenting changes in emotional eating over time from April 2020 (left side) to January 2021 (mid) and May 2022 (right side).

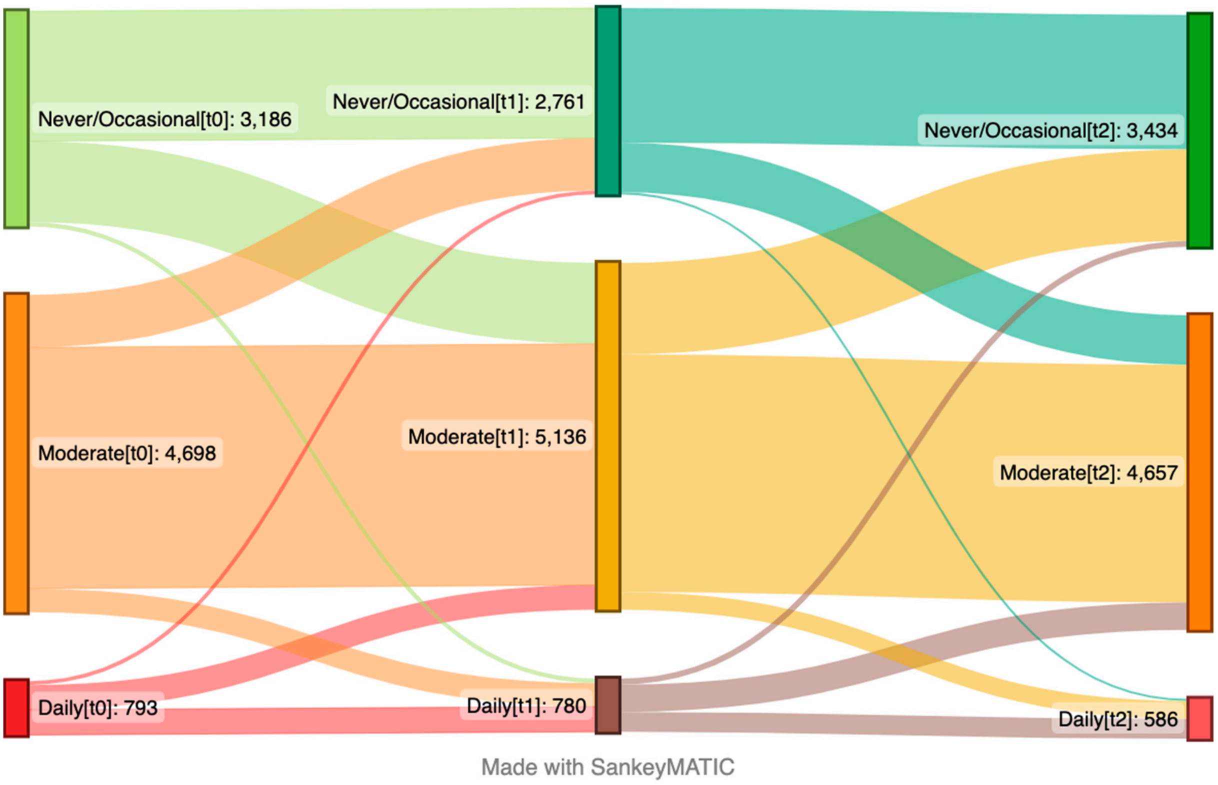

Figure S4. Sankey plot presenting changes in intake of high-sugary food and drinks over time from April 2020 (left side) to January 2021 (mid) and May 2022 (right side). The number in brackets represents time points. This plot includes the participants that answered the items focusing on intake of high-sugary food and drinks on the questionnaire at the first time point (t0), second time point (t1) and third time point (t2) (n=8677).

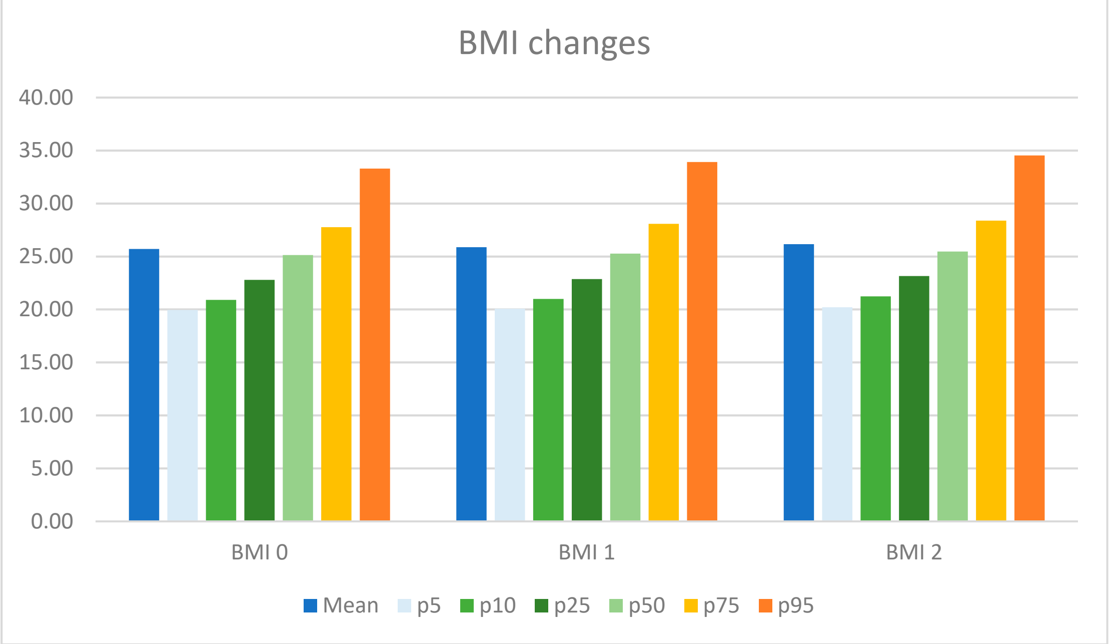

Figure S5. Percentiles of BMI over time.

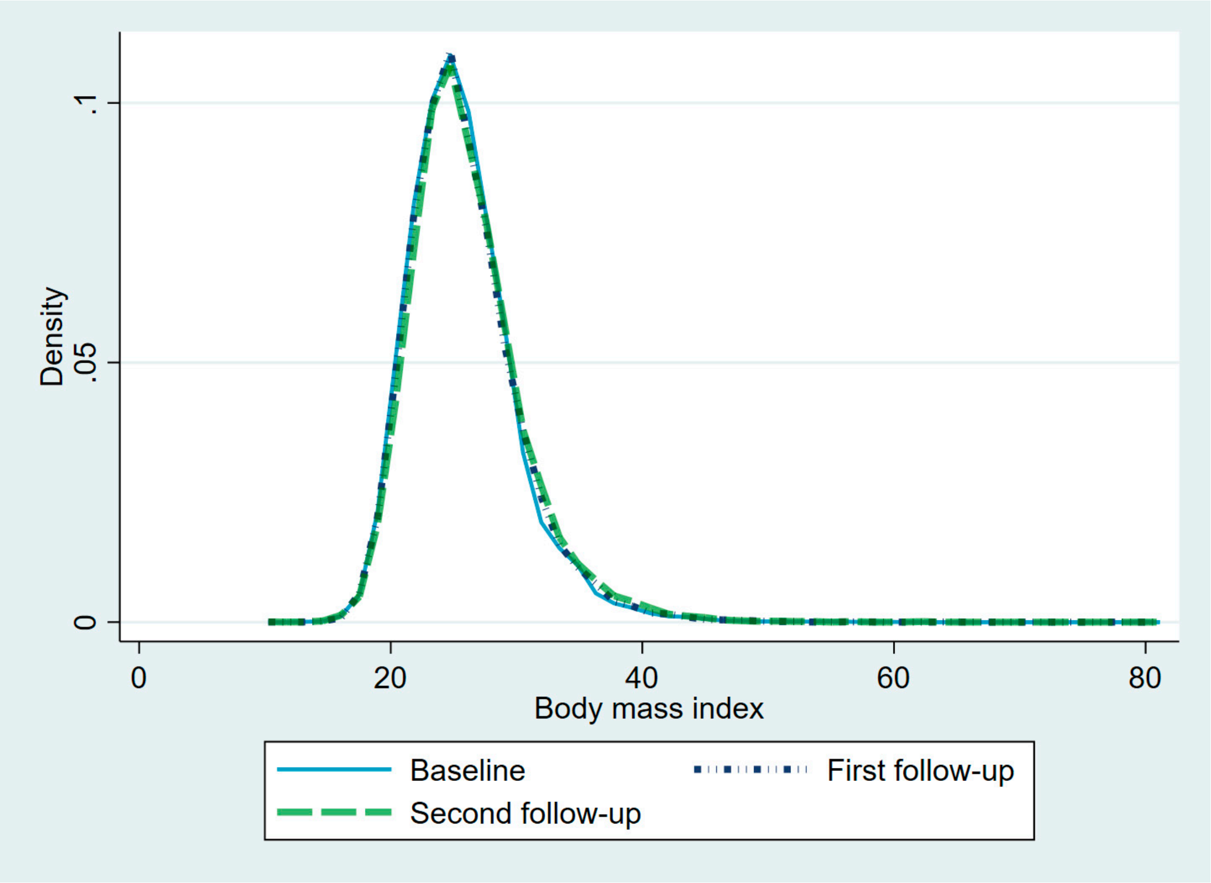

Figure S6. Kernel density for BMI. Comparison of density of BMI in participants in t0, t1 and t2.

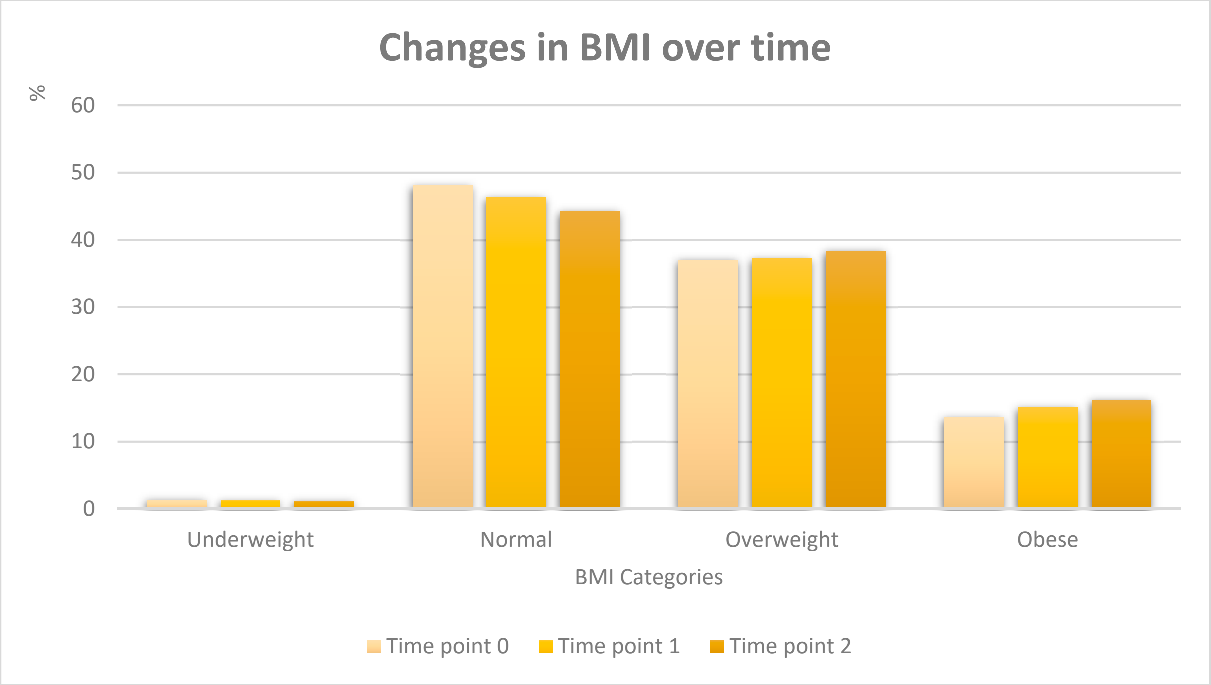

Figure S7. Percentage of participants in BMI categories over time.

Table S3. Mediation of associations between health-related worries and BMI.

Variable	Direct effect	Indirect effect	Total effect	β
**BMI difference**				
Health-related worries	0.14 (0.06 ; 0.23)	0.03 (0.02 ; 0.05)	0.17 (0.09 ; 0.26)	0.03*
Emotional eating	0.06 (0.03 ; 0.09)		0.06 (0.03 ; 0.09)	0.05*
High-sugar foods and drink intake	-0.002 (-0.03 ; 0.02)		-0.002 (-0.03 ; 0.02)	-0.002
Physical activity	-0.02 (-0.08 ; 0.04)		-0.02 (-0.08 ; 0.04)	-0.007
**Emotional eating**				
Health-related worries	0.55 (0.48 ; 0.62)		0.55 (0.48 ; 0.62)	0.17*
**High-sugar foods and drink intake**				
Health-related worries	0.20 (0.13 ; 0.27)		0.20 (0.13 ; 0.27)	0.06*
**Physical activity**				
Psychological distress	-0.04 (-0.07 ; -0.01)		-0.04 (-0.07 ; -0.01)	-0.02*

*P value < 0.05

Table S4. Mediation of associations between financial worries and BMI.

Variable	Direct effect	Indirect effect	Total effect	β
**BMI difference**				
Financial worries	0.11 (-0.01 ; 0.24)	0.05 (0.03 ; 0.08)	0.17 (0.04 ; 0.29)	0.02
Emotional eating	0.06 (0.03 ; 0.09)		0.06 (0.03 ; 0.09)	0.05*
High-sugar foods and drink intake	-0.01 (-0.03 ; 0.02)		-0.01 (-0.03 ; 0.02)	-0.004
Physical activity	-0.03 (-0.03 ; 0.02)		-0.03 (-0.03 ; 0.02)	-0.009
**Emotional eating**				
Financial worries	0.92 (0.82 ; 1.02)		0.92 (0.82 ; 1.02)	0.19*
**High-sugar foods and drink intake**				
Financial worries	0.40 (0.30 ; 0.51)		0.40 (0.30 ; 0.51)	0.08*
**Physical activity**				
Financial worries	-0.04 (-0.09 ; 0.001)		-0.04 (-0.09 ; 0.001)	-0.02

*P value < 0.05

Table S5. BMI and its associations with exposure variables at baseline and over time are presented with coefficients (with 95% confidence intervals). (linear mixed model with inverse probability weighting)

	Fixed effects	Time trend (per year)
Time trends per year		0.16 (0.07;0.25)
Age		
18-40	0 (reference)	0 (reference)
40-60	1.58 (1.45;1.72)	-0.10 (-0.15;-0.05)
60+	1.25 (1.10;1.40)	-0.24 (-0.30;-0.18)
Sex		
Male	0 (reference)	0 (reference)
Female	-1.47 (-1.59;-1.36)	0.07 (0.02;0.11)
Health-related worries		
None or some	0 (reference)	0 (reference)
Substantial	-0.04 (-0.10;0.03)	0.06 (0.01;0.11)
Worries related to economy		
None or some	0 (reference)	0 (reference)
Substantial	0.05 (-0.05;0.14)	-0.02 (-0.12;0.07)
Psychological distress (0=no to 1=extreme)	1.62 (1.18;2.06)	0.20 (-0.03;0.43)
Emotional eating (0=never to 1=everyday)	0.68 (0.54;0.82)	0.27 (0.11;0.43)
High-sugar foods and drink intake (0=no to 1=daily)	0.33 (0.14;0.52)	0.12 (0.00;0.23)
Activity level		
Low	0 (reference)	0 (reference)
Moderate	-0.16 (-0.23;-0.08)	-0.01 (-0.08;0.05)
High	-0.26 (-0.35;-0.18)	-0.07 (-0.13;-0,01)

*linear mixed model presenting absolute coefficients with 0 indicating no difference/change. **Adjusted baseline coefficient of BMI in this model: 25.18 (25.01; 25.35)
